# Continuous intravenous low-dose diclofenac sodium to control a central fever after ischemic stroke in the intensive care unit: a case report and review of the literature

**DOI:** 10.1186/s13256-019-2281-7

**Published:** 2019-12-18

**Authors:** L. G. Giaccari, M. C. Pace, M. B. Passavanti, P. Sansone, V. Esposito, C. Aurilio, V. Pota

**Affiliations:** Department of Women, Child, General and Specialistic Surgery, University of Campania “L. Vanvitelli”, Piazza Miraglia 2, 80138 Naples, Italy

**Keywords:** Central fever, Ischemic stroke, Diclofenac sodium

## Abstract

**Introduction:**

Elevation in body temperature within the first 24 hours of ischemic stroke is fairly common and known to be associated with worse outcomes. Only after thoroughly ruling out infection and the noninfectious etiologies and in the appropriate clinical setting should the diagnosis of central fever be made. Acetaminophen and nonsteroidal anti-inflammatory drugs are typical therapeutic options. External cooling is frequently used when pharmacologic interventions are inadequate. However, reports have suggested that neurogenic fevers are somewhat resistant to traditional pharmacologic therapies.

**Case presentation:**

We describe a case of a Caucasian patient with central fever after ischemic stroke not responsive to acetaminophen administration and external cooling. After an initial bolus of diclofenac sodium (0.2 mg/kg in 100 ml of saline solution for 30 minutes), a continuous infusion (75 mg in 50 ml of saline solution) was started. After 5 days of treatment, the patient’s body temperature was below 37.5 °C, and the diclofenac sodium infusion was stopped.

**Conclusions:**

We observed that a low-dose diclofenac sodium infusion was effective in treating fever without systemic side effects. This treatment may be suggested as an alternative to conventional antipyretic drugs, but additional clinical trials are required.

## Introduction

Fever, defined as a core body temperature exceeding 37.5 °C, is common in patients with brain injury. It most often occurs within the first 2 days after a stroke, and its cause is not always easy to identify. In most cases, infection is the cause of fever after stroke. In severe stroke, massive tissue necrosis and the presence of blood in the brain can elevate body temperature [1].

Fever resulting from a stroke-related pathologic process starts within 24 hours of stroke symptoms, whereas fever of other etiologies emerges at later time points. If infections, venous thromboembolism, drugs reported to cause hyperthermia, and postsurgical origins are excluded, early fever in patients with stroke can indicate a neurological origin [1–3].

Elevation of temperature following stroke is likely the result of metabolic dissociation and heat production related to inflammatory cytokine release in response to the injury [3].

The relationship between fever, neurologic outcome, and stroke size is greatest when the increase in temperature begins within the first 24 hours of neuronal injury [3]. Fever is associated with increased morbidity and mortality in patients with stroke, independent of the temperature elevation origin [1–3].

International guidelines for patients with ischemic stroke recommend treating body temperature higher than 37.5 °C, searching for possible infection, and starting tailored antibiotic treatment. Multimodal cooling and antipyretics, such as acetaminophen, are recommended. The target temperature for patients with neurogenic fever is 37.0 ± 0.5 °C [1].

## Case presentation

A 70-year-old Caucasian woman arrived at the emergency department of a local hospital few hours after a sudden loss of consciousness. Her medical history included type 2 diabetes mellitus being treated with oral hypoglycemic agents and insulin, untreated chronic atrial fibrillation, and transient ischemic attack about 10 years earlier.

On the basis of suspicion of stroke, computed tomographic angiography was performed, which revealed evidence of right middle cerebral artery (MCA) occlusion. Being within 6 hours of stroke onset, intra-arterial thrombectomy was performed, which did not resolve the occlusion. After 7 days of apparent clinical improvement, the patient had a rapid deterioration of the state of consciousness with acute respiratory failure, for which she was intubated, mechanically ventilated, and transferred to our intensive care unit (ICU).

**Fig. 1:**
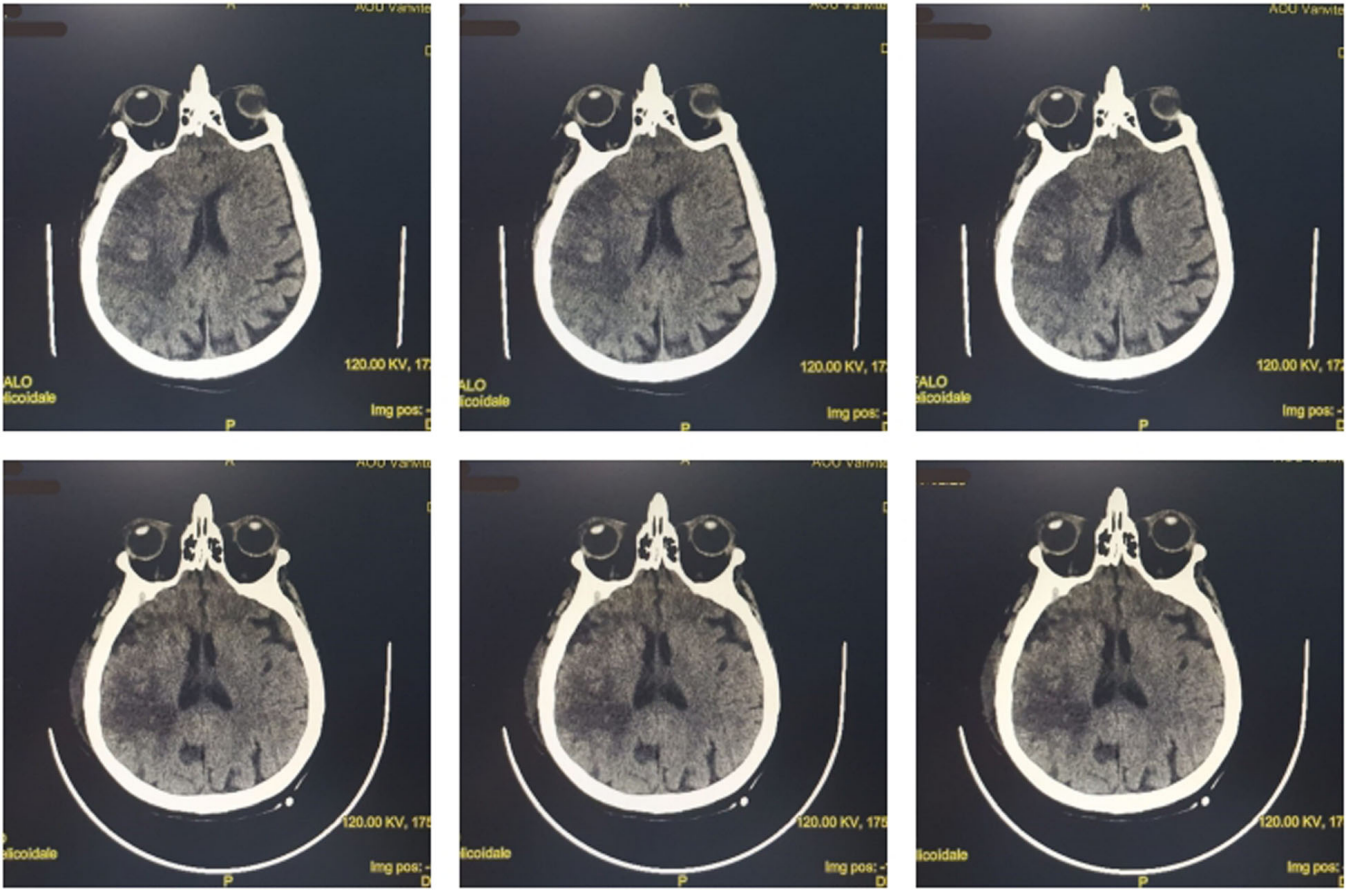
Brain computed tomography (CT). *Top row*: CT images upon arrival at the intensive care unit. *Bottom row*: CT images after 10 days of clinical evolution

At the time of admission, the patient presented with a state of neurological deterioration (Glasgow Coma Scale [GCS] score, 3) and a body temperature of 39 °C. Blood and urine tests, infection investigations, and chest x-ray were performed. Blood tests showed the absence of leukocytosis and negative C-reactive protein (CRP), whereas procalcitonin was not available at the time of admission to the ICU. Tests for urinary, pulmonary, and bloodstream infections required 48 hours for results. No abnormalities were observed on the chest x-ray. Tailored antibiotic treatment was initiated on the basis of suspicion of late-onset hospital-acquired pneumonia with piperacillin/tazobactam 4.5 g intravenously every 6 hours and linezolid 600 mg intravenously every 12 hours. The patient’s fever was treated with acetaminophen 1000 mg up to four times per day, which produced a nonsignificant reduction in body temperature. Brain computed tomography (CT) was performed, which confirmed the presence of an ischemic lesion of the right MCA with involvement of the structures of the right cerebral hemisphere (Fig. 1).

**Fig. 2:**
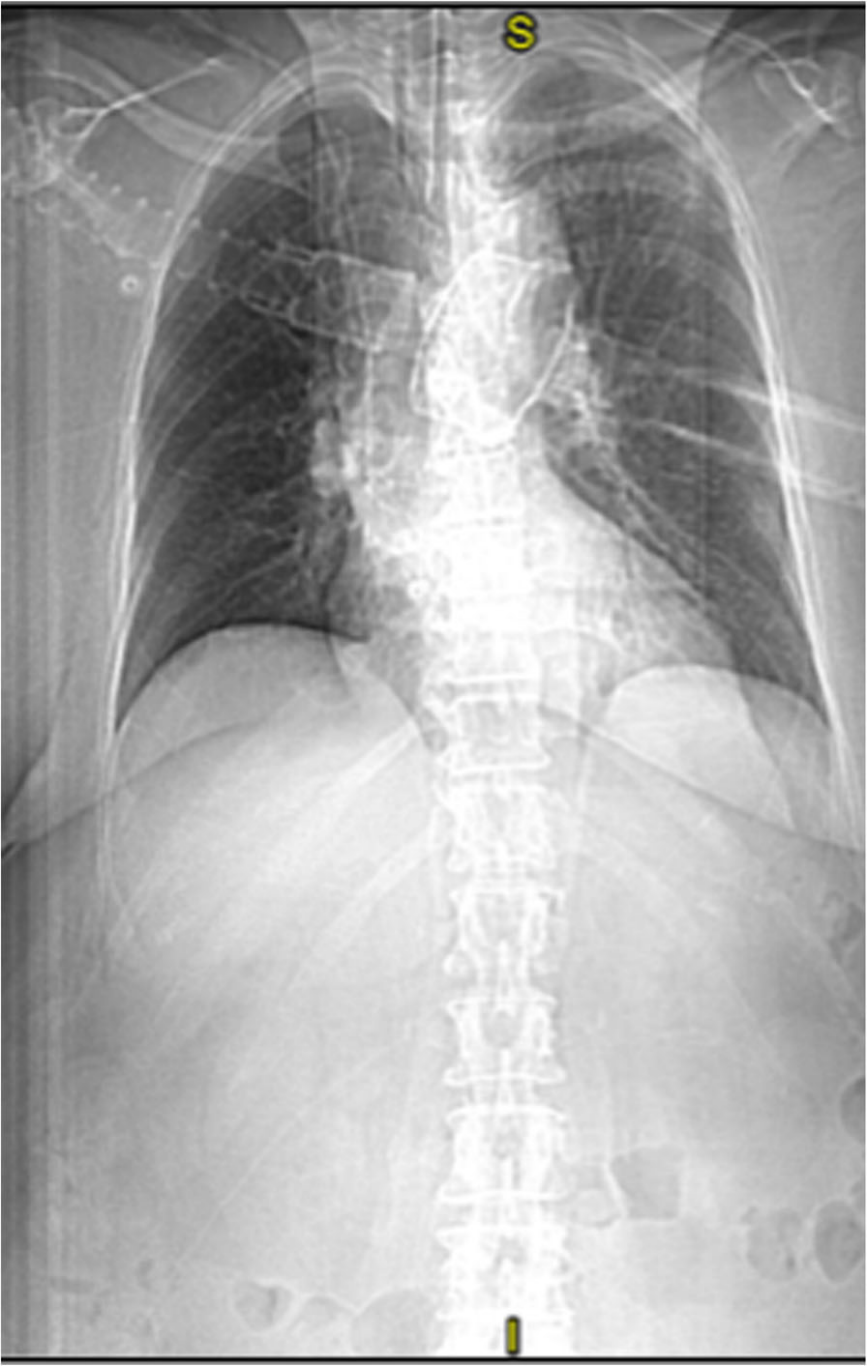
Lung computed tomography

The results of blood, urine, and infection investigations, including bronchial aspirate, were negative, as was the procalcitonin test. Chest x-ray and lung CT did not show signs of infection (Fig. 2).

The antimicrobial therapy was stopped. Despite the administration of acetaminophen and external cooling, there was no reduction in body temperature (maximum body temperature [TC_max_], 40.5 °C) during first 4 days after ICU admission. After thoroughly ruling out infections and the other noninfectious etiologies, the diagnosis of central fever was made. After informing family members and having received written informed consent, a continuous infusion of diclofenac sodium (DFC) was decided. After an initial bolus of DCF (0.2 mg/kg in 100 ml of saline solution in 30 minutes), a continuous infusion (75 mg in 50 ml of saline solution) was started. The dosage of DCF (0.004–0.08 mg/kg/hour) was managed on the basis of the response of body temperature. The DCF infusion was discontinued if the patient’s body temperature was less than 37.5 °C for more than 12 hours with a dose of 0.004 mg/kg/hour.

During the infusion, the patient’s complete blood count, blood pressure, and liver and kidney function were strictly monitored. Continuous monitoring of temperature using an esophageal probe was adopted.

A reduction in body temperature was recorded from the first day of treatment (TC_max_, 38.5 °C), and after 5 days of treatment, the patient’s body temperature was constantly below 37.5 °C, and the DCF infusion was stopped (see Table 1).

**Table 1:** Temperature, heart rate, arterial pressure, and oxygen saturation trends

Day	TC_max_ (°C)	HR (bpm)	Blood pressure (mmHg)	SatO_2_ (%)	Drug
1	39	156	112/60	96	Acetaminophen
2	40	130	106/78	97	
3	40.5	122	114/64	100	
4	40.5	117	101/44	96	
5	39.5	106	136/80	96	Diclofenac
6	38.4	89	122/58	100	
7	37.8	72	154/78	95	
8	37.5	95	117/60	100	
9	37.4	79	117/68	100	
10	37.4	66	115/49	100	–

*Abbreviations: bpm* Beats per minute, *HR* Heart rate, *SatO*_*2*_ Oxygen saturation, *TC*_*max*_ Maximum body temperature

During treatment with DCF, complete blood count, blood pressure, and urinary output were within the normal range. Transient increases in glutamic oxaloacetic transaminaseglutamic (GOT) or aspartate aminotransferase (AST), serum glutamate-pyruvate transaminase (GPT) or alanine transaminase (ALT), and gamma-glutamyl transferase (GGT) were recorded, with immediate normalization after the suspension. Renal function was preserved (creatinine, 0.83 mg/dl; glomerular filtration rate, 72 ml/minute/1.73m^2^).

The patient showed clinical improvement with increased neurological status (GCS score, 9) and hemodynamic stability. Neurological examination revealed evidence of left hemiparesis. During hospitalization, the patient’s neurological status showed no improvement. She died of infectious complications 40 days after admission. The patient’s family members were informed prior to the publication of this article, and they provided written informed consent regarding both the case and the images used.

## Discussion

The main international guidelines (see Table 2) regarding stroke management pay attention to temperature monitoring and management, but no recommendation is made regarding antipyretic treatment [4–7]. In the critical care management of patients with stroke, temperature control appears to have great value [8, 9]. An appreciable reduction in temperature can be achieved by physical cooling, but it can cause chills and vasoconstriction [2]. Surface and endovascular cooling devices can be used. Because fever is caused by a prostaglandin elevation altering thermoregulation control in the hypothalamus, an appropriate intervention is to block this process. Typical antipyretics, including acetaminophen and nonsteroidal anti-inflammatory drugs, are believed to interfere with prostaglandin synthesis. However, reports have suggested that neurogenic fevers are somewhat resistant to traditional pharmacologic therapies; indeed, the use of oral antipyretics to lower body temperature appears inadequate [2]. Acetaminophen in the doses recommended for the treatment of fever has limited efficacy in critically ill patients and often exceeds the maximum daily dose with high risk of liver toxicity [1].

**Table 2:** Temperature management in international guidelines

Society, year	Recommendation
AHA/ASA, 2018 [4]	“Sources of hyperthermia (temperature > 38 °C) should be identified and treated, and antipyretic medications should be administered to lower temperature in hyperthermic patients with stroke.”
HSFC, 2018 [5]	“For temperature greater than 37.5 °C, increase frequency of monitoring, initiate temperature-reducing care measures, investigate possible infection such as pneumonia or urinary tract infection, and initiate antipyretic and antimicrobial therapy as required.”
ESO, 2015 [6]	“In patients with acute ischemic stroke and hyperthermia, we cannot make any recommendation for treating hyperthermia as a means to improve functional outcome and/or survival.”
RCP, 2016 [7]	“Patients with acute stroke should have their clinical status monitored closely, including: level of consciousness; blood glucose; blood pressure; oxygen saturation; hydration and nutrition; temperature; cardiac rhythm and rate.”

*Abbreviations: AHA* American Heart Association, *ASA* American Stroke Association, *ESO* European Stroke Organisation, *HSFC* Heart and Stroke Foundation of Canada, *RCP* Royal College of Physicians

In the literature, eight studies have investigated the use of DCF for fever control in ICU patients with acute brain damage [10–17] (see Table 3). For the treatment of our patient, we relied on the experience of Cormio and Citerio [15]. As already demonstrated, continuous infusion of DCF, even at low doses, provides more stable control of body temperature, minimizing possible adverse reactions. Continuous infusion of DCF was evaluated in two other studies, with similar results [13, 16].

**Table 3:** Characteristics of studies examining antipyretic effects of diclofenac in the intensive care unit

	No. of participants	Dose	Administration route	Infusion duration	Side effects
Zandstra *et al.* [10], 1983	7	100 mg	Rectal	NA	Arterial hypotension, oliguria
Pesenti *et al.* [11], 1986	10	0.2 mg/kg	Intravenous	5 minutes	–
Cormio *et al.* [12], 2000	12	0.04 mg/kg/hour	Intravenous	48 hours	–
Caricato *et al.* [13], 2004	18	0.04 mg/kg/hour	Intravenous	≥ 24 hours	Reduction in MAP
Cormio *et al.* [14], 2004	35	0.17 mg/kg	Subcutaneous	NA	–
Cormio *et al.* [15], 2007	22	0.2 mg/kg (bolus); 0.002–0.08 mg/kg/h (infusion)	Intravenous	Bolus: 30 min Infusion: 6.6 ± 2.3 days	–
Schiefecker *et al.* [16], 2013	21	75 mg	Intravenous	30 min	Reduction in MAP, CPP and PbtO_2_
Picetti *et al.* [17], 2016	30	12.5 mg	Intramuscular	NA	Reduction in HR, MAP, and CPP

*Abbreviations: CPP* Cerebral perfusion pressure, *HR* Heart rate, *MAP* Mean arterial pressure, *PbtO*_*2*_ Brain tissue oxygen tension

Two reports investigated single-shot intravenous use of DCF. In one of the first studies on the subject, DCF 0.2 mg/kg was administered, providing an effective antipyretic effect and avoiding major adverse reactions [11]. In a more recent study, a dose of DCF 75 mg was administered [16]. The authors recorded a reduction in body temperature, but adverse effects were reduced cerebral perfusion pressure and brain hypoxia, both associated with a worse patient outcome.

Other routes of administration have also been evaluated [10, 14, 16]. Low-dose intramuscular DCF is effective for fever control, but it can cause hypotensive episodes that require vasopressor treatment [15].

In our single experience, continuous infusion of DCF allowed normalization of body temperature in the absence of major adverse reactions.

## Conclusions

In conclusion, the development of fever in patients with brain injury admitted to the ICU is a cause of worse clinical outcome. It is essential to diagnose and treat fever in patients with stroke as rapidly as possible. We observed that a low-dose DCF infusion was effective in treating fever, without systemic side effects. This treatment may be suggested as an alternative to conventional antipyretic drugs, but additional clinical trials are required.

## Data Availability

The datasets analyzed during the current study are available from the corresponding author on reasonable request.

## References

[1] Wrotek SE, Kozak WE, Hess DC, Fagan SC. Treatment of fever after stroke: conflicting evidence. Pharmacotherapy. 2011;31(11):1085–91. 10.1592/phco.31.11.1085 PMC3955174 22026396

[2] Meier K, Lee K. Neurogenic fever: review of pathophysiology, evaluation, and management. J Intensive Care Med. 2017;32(2):124–9. 10.1177/0885066615625194 26772198

[3] Gowda R, Jaffa M, Badjatia N. Thermoregulation in brain injury. Handb Clin Neurol. 2018;157:789–97. 10.1016/B978-0-444-64074-1.00049-5 30459041

[4] Powers WJ, Rabinstein AA, Ackerson T, *et al.* 2018 Guidelines for the early management of patients with acute ischemic stroke: a guideline for healthcare professionals from the American Heart Association/American Stroke Association. Stroke. 2018;49:e46–e110. 10.1161/STR.0000000000000158 29367334

[5] Boulanger J, Lindsay M, Gubitz G, *et al.* Canadian Stroke Best Practice Recommendations for Acute Stroke Management: Prehospital, Emergency Department, and Acute Inpatient Stroke Care, 6th Edition, Update 2018. Int J Stroke. 2018;13(9):949–84. 10.1177/1747493018786616 30021503

[6] Ntaios G, Dziedzic T, Michel P, *et al.* European Stroke Organisation (ESO) guidelines for the management of temperature in patients with acute ischemic stroke. Int J Stroke. 2015;10(6):941–9. 10.1111/ijs.12579 26148223

[7] Intercollegiate Stroke Working Party. National clinical guideline for stroke. 5th ed. London: Royal College of Physicians; 2016. https://www.bgs.org.uk/sites/default/files/content/resources/files/2018-06-05/national_guidelines_2016.pdf

[8] McDermott M, Jacobs T, Morgenstern L. Critical care in acute ischemic stroke. Handb Clin Neurol. 2017;140:153–76. 10.1016/B978-0-444-63600-3.00010-6 28187798

[9] Bevers MB, Kimberly WT. Critical care management of acute ischemic stroke. Curr Treat Options Cardiovasc Med. 2017;19(6):41. 10.1007/s11936-017-0542-6 PMC6087663 28466119

[10] Zandstra DF, Stoutenbeek CP, Alexander JP. Antipyretic therapy with diclofenac sodium: observations on effect and serious side effects in critically ill patients. Intensive Care Med. 1983;9(1):21–3. 10.1007/BF01693701 6833624

[11] Pesenti A, Riboni A, Basilico E, Grossi E. Antipyretic therapy in ICU patients: evaluation of low dose diclofenac sodium. Intensive Care Med. 1986;12(5):370–3. 10.1007/BF00292928 3771916

[12] Cormio M, Citerio G, Spear S, Fumagalli R, Pesenti A. Control of fever by continuous, low-dose diclofenac sodium infusion in acute cerebral damage patients. Intensive Care Med. 2000;26(5):552–7. 10.1007/s001340051203 10923729

[13] Caricato A, Conti G, Mercurio G, Mancino A, Santilli F, Antonelli M, Proietti R. Continuous low-dose diclofenac infusion for fever control in patients with acute neurological lesions. Can J Anaesth. 2004;51(9):950–1. 10.1007/BF03018906 15525633

[14] Cormio M, Ergoli C, Citerio G, Portella G, Pesenti A. Subcutaneous diclofenac at low dose is very effective in treating fever with an accompanying reduction in intracerebral pressure in NICU patients [abstract]. Crit Care. 2004;8(Suppl 1):P316.

[15] Cormio M, Citerio G. Continuous low dose diclofenac sodium infusion to control fever in neurosurgical critical care. Neurocrit Care. 2007;6(2):82–9. 10.1007/s12028-007-0002-3 17522789

[16] Schiefecker AJ, Pfausler B, Beer R, *et al.* Parenteral diclofenac infusion significantly decreases brain-tissue oxygen tension in patients with poor-grade aneurysmal subarachnoid hemorrhage. Crit Care. 2013;17(3):R88. 10.1186/cc12714 PMC3706816 23663770

[17] Picetti E, Servadei F, Reverberi C, *et al.* Low-dose intramuscular diclofenac sodium for fever control in acute brain injury. World Neurosurg. 2016;95:241–5. 10.1016/j.wneu.2016.08.032 27544342

